# Family Size and the Age at Infancy-Childhood Transition Determine a Child’s Compromised Growth in Large Families

**DOI:** 10.3389/fped.2022.821048

**Published:** 2022-04-29

**Authors:** Alina German, Lisa Rubin, Galiya Raisin, Ze’ev Hochberg

**Affiliations:** ^1^Department of Pediatrics, Haemek Medical Center, Afula, Israel; ^2^The Ruth and Bruce Rappaport Faculty of Medicine, Technion – Israel Institute of Technology, Haifa, Israel; ^3^School of Public Health, University of Haifa, Haifa, Israel; ^4^Faculty of Medicine, Hebrew University, Jerusalem, Israel

**Keywords:** large family, birth order, childhood height, infancy-childhood transition, child growth

## Abstract

**Background:**

Data on growth of Israeli school children show that children from Jewish ultra-orthodox Haredi and Bedouin Arab families have a higher prevalence of stature below the 3rd percentile. While these populations are usually from lower socioeconomic strata, they also have larger families. This study aimed to evaluate if family structure and the timing of a child’s infancy–childhood transition (ICT) are central to variations in stature.

**Study Design:**

We analyzed the association between family size, birth order and inter-birth interval with child growth and the age at ICT in 3 groups of children, 148 high birth order children from large families (LF ≥ 6), 118 low birth order children from large families (LF ≤ 3) and 150 children from small families (SF).

**Results:**

High birth order children from large families were shorter in childhood than children from small families with a difference of 0.5 SDS in length. We found that birth length and birth order explained 35% of the total variance in infancy length whereas ICT age and infancy length explained 72% of the total variance in childhood length.

**Conclusion:**

Infancy and childhood length are compromised in children from large families. As the family grows larger the younger children tend to be shorter. Reduced length gain in the period between infancy to childhood is when growth is most affected.

## Introduction

Family structure is one of the most important environmental influences on child growth. We have previously shown in a study of preindustrial societies that the inter-birth interval (gestation plus breast feeding period) is a strong indicator of adult size (1). A negative association has been previously reported between birth order and growth rate (2, 3). This finding was reinforced by Moyes, who found that on the island of St. Helena a large percentage of children whose height was less than the 3rd percentile were from families with four or more children (4). Moyes then observed that among families with six or more children, short stature was more prevalent among children with a birth interval of less than 2 years from their younger, but not from their older sibling (5). Studies from New Zealand (6) and Sweden (7) also reported that older children in large families were taller than their younger siblings. A Brazilian study found that this height advantage persisted until early adulthood (8).

**FIGURE 1:**
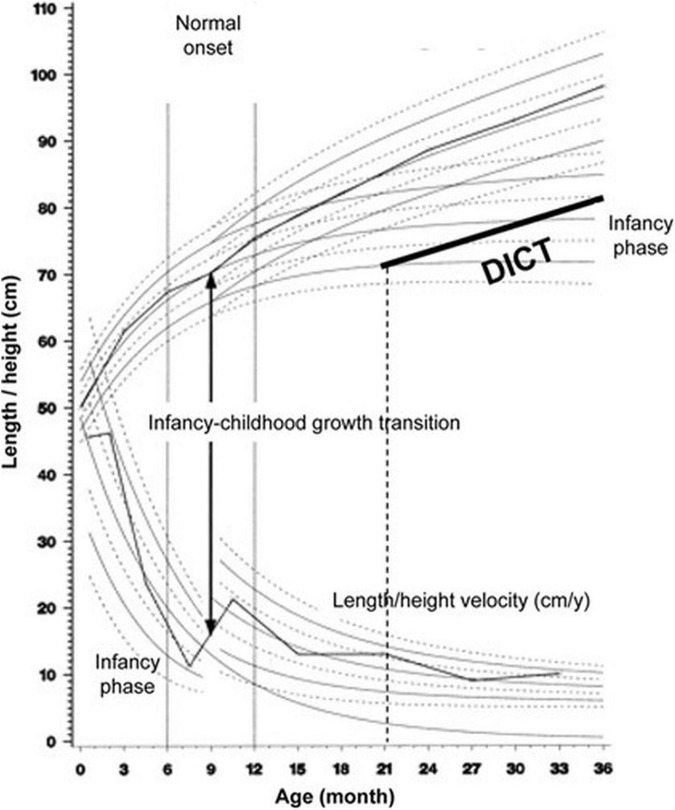
The Infancy, Childhood, and Puberty (ICP) growth model. The ICP growth model divides human growth into three successive and partly superimposed phases that reflect the control mechanisms of the growth process. The infancy phase of the ICP model begins at mid-gestation and tails off at 2–3 y of age. The childhood growth phase sets on in affluent Western countries between 6 and 12 month of age, and when the Infancy to Childhood transition age is delayed (DICT) beyond age 12 month (21 month for this hypothetical child), it has a permanent effect on final adult height. Adapted with permission from Hochberg and Albertsson-Wikland (11).

We propose that Karlberg’s model of human growth is one which may provide insight into the mechanism by which family structure affects growth. The Karlberg’s model describes the infancy, childhood, and puberty (ICP) stages of growth as continuous and overlapping, and defined by transitions driven by sequential additional effects of several endocrine factors that shape the growth trajectory and resultant adult size. Growth during infancy is modulated primarily by nutritional factors whereas during childhood growth hormone sets in as a major regulator of growth (9). The transition from infancy to childhood (ICT) is marked by a growth spurt in length (Figure 1) and has been demonstrated to occur at age 7–12 months in Sweden (10, 11). The ICT correlates negatively and predicts almost 50% of the final adult height variation (11). A delay in ICT has a lifelong impact on stature and is responsible for as many as 44% of children with a normal birth weight and no endocrine disease, who are referred to pediatric endocrine clinics as suffering from “idiopathic” short stature (11). We have previously suggested that a delay in ICT is a predictive adaptational strategy to withstand environmental cues and low energy stores, resulting in short stature (12). In a twin study, we previously showed that the ICT is subject to control by environmental cues such as the intrauterine and household environment (13).

Reports on growth of Israeli school children show that children from Jewish ultra-orthodox Haredi and Bedouin Arab families have a higher prevalence of stature below the 3rd percentile (14). While these populations are usually from lower socioeconomic strata, they also have larger families. We examined the effect of family size and birth order in ultra-orthodox Jewish and Bedouin families (11). This study aimed to evaluate if individual differences in ICT age are adaptive to changes in the family structure and central to variations in stature. To this end, growth of three groups of children was compared: (1) high birth order children (6 and above) from large families (6 or more children), (2) low birth order children (1–3) from large families, and (3) children from small families (1–3 children). Associations between family size, birth order and inter-birth interval with birth, infantile and childhood length, the changes in length standard deviation scores between birth, infancy and childhood and the ICT age were analyzed.

## Materials and Methods

### Subjects

In this retrospective analytical study we studied growth data for children from families whose health records were included in the computerized database of well-child clinics in Israel. This national database is based on the well child health records which began to be computerized stepwise, clinic by clinic, beginning in 1996. Data was collected from 20 clinics that serve primarily Bedouin and Haredi Jewish ultra-orthodox communities where large families tend to prevail. The clinics were in communities whose SES ranking as determined by the Israel Central Bureau of Statistics was among the lowest 3 deciles for the country, with the exception of one clinic in a city with an SES decile of 5. The sample was chosen from children born between 2008 and 2010 and who had older siblings with longitudinal growth data recorded in the computerized records. The infants were measured for body weight and length after birth and then during the first 36 months of their life at their routine follow-up visits by trained nurses, using standardized and periodically calibrated equipment. Only children whose records contained >3 length measurements before age 10 months (the average ICT age in Israel) and >3 length measurements between ages 12–36 months were included in the study. Exclusion criteria included families with twin siblings, childhood length/height ≤−3 SD (*n* = 2).

Information on birth date, gender, gestational age, birth weight and length, ethnicity, family size at the time of birth, birth order, interval in days from the previous and subsequent child birth and height measurements from birth until the age of 36 months was obtained from the individual health record of the participants.

Initially 657 children answered the inclusion criteria and were selected for evaluation. The data were not clustered by family. Excluded from the analysis were: 161 children for whom it was not possible to determine their ICT, an additional 78 children for whom the difference in their ICT as determined by the 2 readers was greater than 2 months and 2 children with birth orders ≥10. Altogether, 416 (70%) of the initially selected children were included in the analysis.

The study included 416 subjects who were divided into three groups according to family size and birth order of the child: 148 high birth order (6 or greater) children from families with six or more children (LF ≥ 6), 118 low birth order children (3 or less) from families with six or more children (LF ≤ 3), and 150 children from small families with 3 or fewer children (SF). The summary of birth data of subjects is presented in the Table 1.

**TABLE 1:** Baseline characteristics for all children included in the study and comparison of the main study variables between the study groups.

Characteristics	LF ≥ 6 *n* = 148	LF ≤ 3 *n* = 118	SF *n* = 150	χ2	*T*-test
				*p*-value	*p*-value
Gender (% male)	50	44.9	53	0.391	
Ethnicity (% Jewish)	14.9	20.3	22	0.264	
SGA (%)	13.5	21.2	18	0.249	
Birth Weight preterm mean, (SD)	2.62 (0.28)	2.24 (0.32)	2.91 (0.37)		0.141
Birth Weight term mean, (SD)	3.36 (0.4)	3.23 (0.44)	3.29 (0.41)		
Gestational Age (% < 37 weeks)	4.7	0.8	2.7	0.168	
Family size (number per family size)					
3	–	–	150		
6	41	39	–		
7	58	43	–		
8	30	19	–		
9	11	9	–		
10	7	6	–		
Birth order (number per birth order)			–		
1	–	38	33		
2	–	46	71		
3	–	34	46		
6	87	–	–		
7	43	–	–		
8	14	–	–		
9	4	–	–		
IBIp in years (mean, SD)	2.5 (0.83)	1.74 (1.08)	3.07 (1.84)		**<0.001**
IBIn in years (mean, SD)	2.58 (1.16)	1.55 (0.57)	3.57 (1.9)		**<0.001**

*Baseline characteristics of children included into the study groups. LF – large families with 6 or more children, LF ≥ 6 – children with birth order ≥6 from large families; LF ≤ 3 – children with birth order ≤3 from large families; SF ≤ 3 – small families with 3 children or less. SGA- children born small for gestational age. IBIp-interbirth interval in years from the previous child birth to the child birth. IBIn-interbirth interval in years from the current child birth to the next child birth. Chi square, t-test. Significant results presented in bold, p < 0.05.*

Since not all infants had length recorded at birth, the first length measurement noted in the record, was provided and defined as a birth length, provided it was within 2 weeks after birth. Since in Israel, the mean age of the ICT is 10.9 months (13), we defined the last length measurement closest to the age of 7 months as infantile length. The last length/height measurement closest to the age of 24 months was defined as childhood height. Length/height measurements were Z-transformed using the World Health Organization (WHO) Anthro program. The difference between infantile (last measurement before age 7 months) and birth length SDS was defined as ΔZ_1_. The difference between childhood length SDS (last measurement before age 24 month) and infantile length SDS was defined as ΔZ_2_, The difference between birth length SDS and the childhood length SDS was defined as ΔZ_3_.

The study was approved by the ethics committee of the Israeli Ministry of Health.

### Infancy-Childhood Transition Determination

The data collected in the first 2 years of life of each subject were fitted using a mathematical model that incorporated the functions and concepts of the Infancy and Childhood components of Karlberg’s infancy-childhood-puberty (ICP) growth model (15), as previously described (13, 16–18). The infancy component is modeled with a negative exponential function: Y = a_I_ + b_I_(1 − exp(-c_I_t)], where the birth length is aI, the postnatal contribution of the infancy component is bI, and the growth rate of the infancy component is c_I_. The childhood component is represented by a second-degree polynomial function, where t is age in years: Y = a_C_ + b_C_t + c_C_t.^2^ All measurements were automatically plotted on the ICP growth model charts (19). The age of the Infancy-Childhood Transition (ICT) was determined to the nearest month by visual inspection of the plots by two observers (inter-observer coefficient of variation, CV < 1 month, *n* = 100), as previously described (16, 17, 20).

### Statistical Analysis

Sample size calculation: in order to detect a difference of 0.50 SDS between length SDS at birth and that in childhood, with an alpha error of 5% and a beta error of 80%, a sample of 377 was needed. All statistical analyses were done using a software statistical package (IBM SPSS Statistics 20.0) and statistical significance was set as 5%.

Data are presented as percent incidence for categorical variables and the mean ± SD for continious measurements. For comparison between the study groups we used the chi square test to assess the relationship between categorical variables (sex, ethnicity, percent of children born small for gestational age), and the *T*-test and ANOVA for comparison between continious variables (birth weight of children born preterm and term and the interval from the previous and the next child birth), and for comparison between the study groups on birth, infancy and childhood height, ΔZ_1_, ΔZ_2_ and ΔZ_3_, ICT age, infancy and childhood weight. The distribution of the variables was normal; the skewness did not exceed 0.19 and the kurtosis did not exceed 1.2. The Pearson’s correlation coefficient was used to calculate the strength of the linear relationship between birth order and family size and study variables and also between childhood height and ICT age. Multi linear regression analysis was used to distinguish between different independent factors that form the variation of the infancy length and childhood height expressed as SDS. The restricted model (with the minimal number of the estimated variables), whose performance did not differ significantly from the general model, was considered as the best fitting model.

## Results

No differences were found in gender and gestational age between the three study groups. The percent of preterm children and those born small for gestational age was comparable between the 3 study groups. Birth weight for term born infants was highest among higher birth order infants from large families, but the difference was not significant between the groups. 266 children (63%) had 6 or more siblings (Table 1). The interbirth interval from the previous sibling and from the subsequent sibling, was significantly shorter for all children from large families (*p* < 0.001 for both) than for those from small families.

### Growth Pattern

Children from small and large families had comparable birth length, however by childhood (age 2 years) children from large families were shorter than children from small families. There was a difference of 0.5 SDS in length between high birth order children in large families (LF ≥ 6) and children from small families (SF). High birth order children from large families “lost” 0.1 ± 1.0 SDS during infancy and an additional 0.2 ± 0.6 SDS between infancy to childhood (Table 2). Specifically, childhood length was 0.7 ± 0.9 SDS for LF ≥ 6 vs. −0.2 ± 0.9 for SF (*p* < 0.001). Low birth order children from the large families had a greater loss of childhood length than comparable birth order children from small families: they lost 0.3 ± 0.8 SDS in length during transition from infancy to childhood and their childhood length was slightly shorter (−0.4 ± 1.0 SDS) than that of children from small families (–0.2 ± 0.9 SDS, *p* < 0.04).

**TABLE 2:** Comparison of the main study variables between all children included in the study.

	High birth order-large families	Low-birth order-large families	Small families	*p*-value LF ≥ 6 vs. LF ≤ 3	*p*-value LF ≥ 6 vs. SF	*p*-value LF ≤ 3 vs. SF
Birth Length SDS (sd)	−0.30 (1.06)	−0.36 (0.99)	−0.16 (1.00)	0.856	0.504	0.253
Infancy Length SDS (sd)	−0.41 (0.82)	−0.12 (1.03)	−0.07 (1.01)	**0.032**	**0.004**	0.915
Childhood Length SDS (sd)	−0.66 (0.86)	−0.44 (0.96)	−0.16 (0.94)	**0.128**	**<0.001**	**0.038**
Infancy weight SDS (sd)	−0.10 (0.94)	−0.10 (0.97)	0.14 (0.98)	0.67	**0.026**	0.081
Childhood weight SDS (sd)	0.14 (0.89)	0.03 (0.92)	0.36 (0.92)	0.36	**0.049**	**0.002**
ΔZ1	−0.12 (0.96)	0.25 (0.86)	0.10 (0.91)	**0.004**	0.109	0.388
ΔZ2	−0.25 (0.63)	−0.32 (0.77)	−0.09 (0.76)	0.675	0.167	**0.029**
ΔZ3	−0.36 (1.08)	−0.08 (1.06)	0.00 (1.01)	0.070	**0.008**	0.814

*Birth length, infancy (age 7 m) length, childhood (age 2 y) height in SDS and length SDS change between birth to infancy (ΔZ1), infancy to childhood (ΔZ2) and birth to childhood (ΔZ3). LF – large families with 6 or more children, LF ≥ 6 – children with birth order ≥6 from large families; LF ≤ 3 – children with birth order ≤3 from large families; SF ≤ 3 – small families with 3 children or less. Presented as Mean and Standard deviation (SD). ANOVA test, Post-hoc tests. Significant results in bold, p < 0.05.*

Within large families, infancy and childhood weight SDS was comparable between high and low birth order children; specifically infancy weight SDS was −0.1 ± 0.9 for LF ≥ 6 vs. −0.1 ± 1.0 for LF ≤ 3, (NS) and childhood weight SDS was 0.1 ± 0.9 for LF ≥ 6 vs. 0.0 ± 0.9 for LF ≤ 3, (NS). LF ≥ 6 children had slightly lower weight SDS in infancy than children from SF −0.1 ± 0.9 vs. 0.1 ± 1.0 (*p* = 0.03) and the weight SDS difference between these two groups in childhood increased 0.1 ± 0.9 vs. 0.4 ± 0.9, (*p* = 0.05).

### Infancy-Childhood Transition Age

Infancy-Childhood Transition age for all study subjects was on average 11.9 ± 1.8 months and was comparable between 3 study groups. The variance for ICT among children from large families was greater than that for children from small families (2.97 LF ≥ 6 vs. LF ≤ 3 3.98 vs. SF 2.77.

### Multiple Linear Regression Analysis

A summary of results of the multiple linear regression analysis for the infantile and childhood lengths SDS for the study cohort are shown in Tables 3, 4. In general, 34.5% of the total variance in infancy lengths SDS (age 7 months) was explained by the independent factors included in the model (*R*^2^ = 0.35, *p* < 0.0001), (Table 3). We found a substantial contribution of birth length SDS (ß = 0.56, *p* < 0.001) and birth order (ß = −0.16, *p* < 0.0001). As expected, infancy length was inversely correlated with birth order. Our model showed no significant contribution of the birth weight, gestational age and family size to interindividual variation in infantile length SDS.

**TABLE 3:** Multivariate regression analysis of infancy length SDS.

	B	Std. error	Beta	*p*-Value	95.0% Confidence Interval for Beta
(Constant)	0.188	0.073		**0.010**	0.046; 0.331
**Birth Length**	0.534	0.038	0.566	**0.000**	0.460; 0.608
Gestational age	0.041	0.032	0.051	0.196	−0.021; 0.103
Birth Weight	0.166	0.119	0.073	0.165	−0.069; 0.401
Family Size	−0.005	0.020	−0.011	0.810	−0.045; 0.035
**Birth Order**	−0.068	0.017	−0.163	**0.000**	−0.101; −0.036

*Factors associated with the Infancy length (7 months). R^2^ = 0.35, p < 0.0001. Significant results are displayed in bold numbers.*

**TABLE 4:** Multivariate regression analysis of childhood height SDS.

	B	Std. error	Beta	*p*-Value	95.0% Confidence Interval for Beta
**(A) Factors associated with Childhood height (2 years) ICT age included.**					

(Constant)					
Birth Length	0.050	0.034	0.054	0.143	−0.017; 0117
Gestational age	0.013	0.020	0.018	0.528	−0.027; 0.053
Birth Weight	0.064	0.077	0.029	0.409	−0.088; 0.215
Family Size	–0.016	0.013	–0.037	0.240	−0.042; 0.011
Birth Order	–0.025	0.013	–0.061	0.056	−0.051; 0.001
ICT age	–0.244	0.014	–0463	**0.0001**	−0.272; −0.216
Infantile Length	0.699	0.032	0.706	**0.0001**	0.627; 0.751

**(B) Factors independently associated with Childhood height (2 years) ICT age excluded.**					

(Constant)	0.144	0.328		0.661	−0.500; 0.788
Birth order	–0.007	0.017	–0.017	0.685	−0.040; 0.026
Family size	–0.054	0.017	–0.127	**0.002**	−0.0087; −0.02
Birth weight	–0.027	0.097	–0.012	0.780	−0.217; 0.163
Birth length	0.028	0.045	0.030	0.533	−0.060; 0.115
Infantile length	0.657	0.041	0.673	**0.000**	0.575; 0.738

*R^2^ = 0.72, p < 0.0001. R^2^ = 0.52, p < 0.0001. Significant results are displayed in bold numbers.*

Almost 75% (*R*^2^ = 0.72, *p* < 0.0001) of the variance in childhood length SDS (age 2 years) was explained by the model (Table 4A) that included three independent factors: infancy length SDS (ß = 0.71, *p* < 0.0001), ICT age (ß = −0.0.46, *p* < 0.0001), and birth order (ß = −0.63, *p* = 0.048). Delayed ICT age and higher birth order were inversely associated with childhood length. Our model showed no significant contribution of birth weight, birth length, and family size to childhood length.

When multiple regression analysis was done for the childhood lengths as a function of the same variables as in previous analysis, but excluding ICT age (Table 4B), the model explained only 52% of the variance (*R*^2^ = 0.52, *p* < 0.0001) and included two independent factors: infancy length SDS (ß = 0.67, *p* < 0.0001) and family size (ß = −0.13, *p* = 0.002).

## Discussion

The study was designed to evaluate the effect of family size, birth order, birth intervals and ICT age on growth. Our novel study design allowed comparison of lower and higher birth order children from the families as well as to comparable birth order children from smaller families of similar socioeconomic backgrounds. We assumed that even in large families, the first three children spend their infancy in a small family, and we could therefore evaluate the role of the birth rank and family size on growth. However we point out that the large families differ from small families from the outset as the interbirth interval among these families is shorter even for the low birth order children. We confirm suggestions from previous studies that children in large families are shorter than those in smaller families (3, 4). However in the separate families group analysis we found that birth order and family size were negatively associated with infant and childhood length as well as length gain from birth to infancy and from birth to childhood. The strength of this association increased with age. We previously proposed that adult size is determined to a great extent during the transition from infancy to childhood (11), and demonstrated in a twins study that of the ∼50% of the variance provided to adult height by the ICT, 42.2% is due to adaptive cues represented by shared twin and sibling environment, with no detectable genetic involvement (13).

The Infancy-Childhood-Puberty model of human growth proposes distinct phases of human growth within the context of hormonal regulation of growth (19). The model has been incorporated into the evolutionary life history theory (21) to explain environmental cues and influences on growth hormone (GH) activation at the time of the ICT (9). Infants with a somewhat compromised nutritional supply might delay the onset of GH activation as a strategy to preserve energy-demanding brain growth in the early years of life. The average age of the ICT in this study was delayed by 1.5 months compared to that previously published for Israeli children born to higher SES families from the Tel Aviv and Haifa districts (13). This finding supports the known significant effect of SES on childhood growth.

In the present study birth order was an independent factor affecting the length at age 7 months (infancy) whereas for childhood length (age 2 years) family size became a significant factor. Family size and infancy length were the significant factors explaining 52% of the variance in childhood length in the regression models. However when ICT age was included in the regression model, it became a leading factor, while family size lost its significance. The model that included ICT was the most fitted, explaining 72% of the variance. Thus, even though the ICT age was comparable between the three study groups in our study, this variable became a leading independent factor affecting childhood height in the multiple regression analysis We note that the variance for the ICT was greater among the children from larger families than for those from small families. It is possible that effect of the ICT in the regression model might reflect either a direct effect or is due to covariance with other unmeasured variables.

We suggest that delayed ICT is a possible mechanism by which the large family size affects child growth resulting in their smaller stature (11). The difference between childhood and infancy length (ΔZ_2_), which is established through a delayed ICT, was greater than the difference between infancy and birth length (ΔZ_1_).

The mechanisms responsible for signaling the ICT are as yet unresolved. Resource dilution whereby children in larger families endure disadvantage by having to share limited resources among more children has been suggested as the mechanism by which growth and other outcomes of children in large families are hampered. Specifically, nutritional disadvantage may provide another explanation for the slower growth of children in large families. Some studies have found that the overall nutritional value of the food provided to children in larger families may be inferior to that provided in smaller families and consequently fail to meet the children’s nutritional requirements (22). Other findings, however suggest that undernutrition may not be the central mechanism for the findings here. If poverty compromised the quality of diet in large families, we would expect that children higher up the family birth order be more adversely affected than those born earlier. In the British Millenium study (23), babies who came mostly from Jewish Haredi large families (54% had 5–12 siblings) had greater weight faltering (weight mean SDS difference −1.1) than height SDS loss by age 1 year (length SDS difference −0.5). The difference between Israeli and British Haredi families may lie more in family size and emotional stress than the family economy (24).

Increased infectious load provides another possible explanation for the differential growth and maturation of infants from large families. Young infants in larger families often suffer from frequent colds and minor infections, usually brought home from school by the older children. Gibson and McKeown (25), studying morbidity in the first year of life in Birmingham, say that “it seems reasonable to suppose that the increased risk to later births in poor circumstances is due, at least in part, to increased infection conveyed by older siblings” (25).

An analysis of data collected by the Avon Longitudinal Study of Parents and Children found that a significant decrease in the amount of care that both mother and father give to each child with the advent of each additional sibling (26). Apart from SES and parental education, family size was the most important variable explaining measures of parental care. Another analysis by the same researchers found an effect of family size upon growth at age 10, most significantly among higher birth order children (26). In a recent study, children living in larger households (≥6) were 1.5 times as likely to be neglected by their parents as were children living in smaller households (<6). There is ample evidence showing that the ICT is a function of the switch from Growth Hormone (GH)-independent to GH-dependent growth (11, 15, 17). The role of emotional deprivation in growth hormone secretion is well-established, from the initial observation that emotional deprivation and growth retardation simulate hypopituitarism (27), to studies of GH levels before and during catch-up growth in emotional deprivation and shorter stature (28) and up to a recent study showing growth failure associated with early neglect in United States children and international adoptees (29). We thus suggest that shorter stature in large Haredi and Bedouin families may stem from competition for finite parental attention and resources which translate into delayed activity of the GH-IGF axis resulting in delayed ICT and a consequential loss of length in the transition from infancy to childhood. It would seem that the stronger inverse relationship between length gain and family size found in our study as the child approaches age 2 years is associated with the new competition starts with the birth of the subsequent child.

Alternatively, intergenerational effects could account for the lesser growth seen in the children of the large families.

### Limitations

As described above, determination of the ICT is visual and dependent upon the number and timing of measurements. In the present analysis, it was impossible to determine the ICT for almost a quarter (161/657) of the study children. Recently it was proposed to use the infancy-childhood transition of weight for age *z*-score, based on the Widdowson and Cambridge Infant Growth Study (CIGS) (30, 31), however this model requires validation and endocrine and evolutionary collateral.

The data was retrospectively retrieved from the personal health records of the children in well baby clinics and the information on the parental height, parental education and child rearing practices were not available in the records, so the effect of these factors on the length/height at 2 years could not be included in the analysis.

Another limitation stems from the inherent difficulty in separating out the effect of SES from that of family size. Family size is closely associated with SES. We collected our data from well-baby clinics in towns with high proportions of ultra-orthodox and Bedouin families, known to have larger families but also to have lower SES (32, 33). Berman in his economist’s view on ultra-orthodox Jews, demonstrated that in mid-90s average ultra-orthodox family was large with 4.5 children at home, their monthly income was 42% of the income of the average two parent Israeli family which supported only 2.1 children. The Bedouin Arab population (34) has the lowest socioeconomic level of any population group in Israel (35). Another caveat stems from possible misclassification of family size among the research families; family size was determined by the information in the health record at the time of data extraction and it is possible that this was not the final family size.

Despite these inherent limitations, we suggest that our findings support the hypothesis that family size, birth order and delayed ICT affect childhood growth. Our study design which permits the comparison of children of similar SES to examine the effects of increasing family size and higher birth order corroborates the findings of Lawson and Mace in British families regarding these 2 factors (26).

This study demonstrates that family size affects child growth and that ICT is a central mechanism for shorter stature in large families and is responsible for the early onset of this effect.

Since the data set for the study was extracted directly from the personal health records of the children, the Ministry of Health does not allow sharing of the raw data, even though de-identified. The aggregated data are not publicly available but are available from the corresponding author on reasonable request with publication of the paper.

## Data Availability Statement

The datasets presented in this article are not readily available because the study was extracted directly from the personal health records of children. Although deidentified, the Ministry of Health does not allow sharing of the raw data. Requests to access the datasets should be directed to LR, lisa.p.rubin@gmail.com.

## Ethics Statement

The studies involving human participants were reviewed and approved by the Ethics Committee of the Israeli Ministry of Health. Written informed consent for participation was not required for this study in accordance with the national legislation and the institutional requirements.

## Author Contributions

AG co-conceptualized and co-designed the study, co-analyzed the data, drafted the manuscript, revised the final version critically for important intellectual content, and approved it for publication. LR co-conceptualized and co-designed the study, co-analyzed the data, revised the manuscript critically for important intellectual content, and approved it for publication. GR collected the data, co-analyzed and interpreted it, revised the manuscript critically for important intellectual content, and approved it for publication. ZH conceptualized and designed the study, supervised the data collection, co-analyzed the data, drafted the initial manuscript, reviewed and revised the manuscript, and approved it for publication. All authors approved the final manuscript as submitted and agreed to be accountable for all aspects of the work.

## Conflict of Interest

The authors declare that the research was conducted in the absence of any commercial or financial relationships that could be construed as a potential conflict of interest.

## Publisher’s Note

All claims expressed in this article are solely those of the authors and do not necessarily represent those of their affiliated organizations, or those of the publisher, the editors and the reviewers. Any product that may be evaluated in this article, or claim that may be made by its manufacturer, is not guaranteed or endorsed by the publisher.
